# The Prevalence of Pulmonary Hypertension Among Maintenance Dialysis Patients With ESRD and Its Associated Factors: A Retrospective Study

**DOI:** 10.3389/fmed.2020.570874

**Published:** 2020-12-04

**Authors:** Ying Zhang, Xiao-Han Ding, Rongsheng Rao, Yiqin Wang, Fang Pang, Sha Tang, Ling Nie, Shi-Zhu Bian

**Affiliations:** ^1^The Key Laboratory for the Prevention and Treatment of Chronic Kidney Disease of Chongqing, Department of Nephrology, Kidney Center of People's Liberation Army, Xinqiao Hospital, Army Medical University (Third Military Medical University), Chongqing, China; ^2^Department of Health Care and Geriatrics, The 940th Hospital of Joint Logistics Support of People's Liberation Army, Lanzhou, China; ^3^Department of Ultrasonography, Xinqiao Hospital, Army Medical University (Third Military Medical University), Chongqing, China; ^4^Institute of Cardiovascular Diseases of People's Liberation Army, Xinqiao Hospital, Army Medical University (Third Military Medical University), Chongqing, China; ^5^Department of Cardiology, Xinqiao Hospital, Army Medical University (Third Military Medical University), Chongqing, China

**Keywords:** prevalence, pulmonary hypertension, maintenance dialysis, end-stage renal disease (ESRD), a retrospective study

## Abstract

**Aim:** To determine the prevalence of pulmonary hypertension (PH) and its associated factors among end-stage renal disease (ESRD) patients who underwent maintenance dialysis.

**Methods:** A total of 491 patients received echocardiography examinations and underwent pulmonary artery systolic pressure (PASP) assessments. A subgroup of 283 patients were subjected to plasma creatinine (Cr) and blood urea nitrogen concentration (BUN) tests, routine blood examinations and electrolyte analysis. First, we compared the differences in echocardiographic, Cr and BUN, blood routine and electrolyte parameters between PH and non-PH groups. The correlations between PASP and the parameters mentioned above were also analyzed. Furthermore, univariate and adjusted logistic regression analyses were performed to identify the independent associated factors.

**Results:** The incidence of PH among ESRD patients who were treated with maintenance dialysis was 34.6%. Most of the echocardiographic parameters, including end-diastolic internal diameters of the left atrium, left ventricle, right atrium, and pulmonary artery, as well as interventricular septum mobility, left ventricular posterior wall mobility, fractional shortening, stroke volume and left ventricle ejection fraction (LVEF), were associated with PH. Furthermore, Mg^2+^ (*p* = 0.037) and Cl^−^ (*p* = 0.043) were significantly associated with PASP. However, after adjustments were made in the regression analysis, only internal diameters of the left atrium, right atrium, and LVEF were independently associated with PH.

**Conclusion:** PH is prevalent, with a relatively high incidence among ESRD patients who undergo maintenance dialysis. The sizes of the left and right atria as well as LVEF were independently associated with PH, but further cohort and basic mechanistic studies are needed to confirm this finding.

## Introduction

Pulmonary hypertension (PH) is reported to be one of the most frequent complications among various diseases including end-stage renal disease (ESRD) and is characterized by progressively increased pulmonary arterial pressure (PAP) (1–4). PH is the leading cause of right heart failure among cardiovascular diseases (1, 5, 6). Additionally, PH is recognized to be a novel threat for ESRD patients (7). It is reported that the incidence of PH is between 9 and 39% among patients with stage 5 chronic kidney disease, this value ranges from 8.8 to 68.8% among ESRD patients who undergo hemodialysis (2). Whilst, the incidence of PH is up to 42% among peritoneal dialysis-treated patients (8–11).

However, the associated risk factors and precise mechanisms underlying PH in ESRD patients have not been specifically addressed. Some researchers have suggested that the volume overload, severe anemia, hemodynamic modifications, hyperparathyroidism induced by renal failure may be involved in the development of PH (4, 10, 12, 13). Left heart dysfunction in ESRD patients has also been considered to be the underlying cause of PH (14). Furthermore, the respiratory system related diseases such as chronic obstructive pulmonary disease have also been indicated to the important causes for PH.

Although a few previous studies have reported some risk factors for PH in ERSD which belong to the V classification PH (PH with multiple-reason/unclear mechanisms/ caused by renal dysfunction) (15). This kind of PH may be caused by toxicity of the metabolic wastes which cannot been excreted in renal failure patients (16, 17). Furthermore, the blood components and electrolytes may be also the causes of PH (18). There are still many factors have not been reported in renal failure patients with PH.

Thus, we conducted this retrospective study with a large size of population from March 2011 to June 2019 in our dialysis center to determine the prevalence of PH and its associated factors.

## Methods

### Study Design and Population

**Figure 1:**
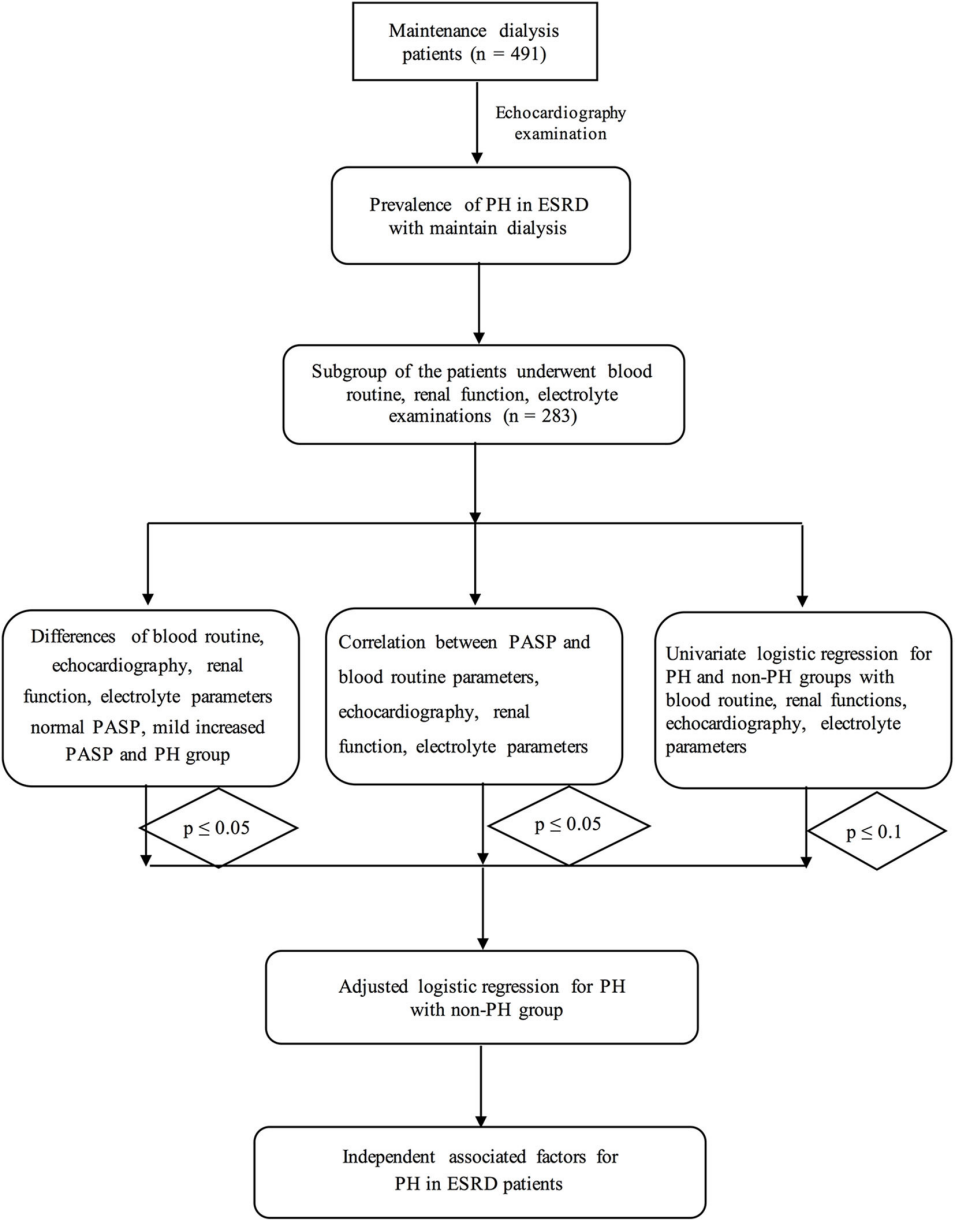
The flow chart of this study.

This was a retrospective study of patients with ESRD who underwent maintenance dialysis from March 2011 to June 2019 in the Nephrology of Chongqing and Kidney Center of PLA, Xinqiao Hospital, Army Medical University (Third Military Medical University). A total of 491 ERSD patients who underwent hemodialysis were selected for this study and underwent echocardiography examination according to the inclusion and exclusion criteria. Exclusion criteria included: previous pulmonary embolism, collagen vascular disease, obstructive sleep apnea, chronic obstructive pulmonary disease (COPD) as well as the moderate/severe mitral or aortic valve diseases. Furthermore, patients with liver failure or heart failure have also been excluded. A subgroup of 283 patients received Cr and BUN tests, routine blood examinations and electrolyte analysis. The procedure is shown in Figure 1.

Each patient was completely informed of the purpose and procedure of the study and provided written informed consent. The current study was designed in compliance with the Declaration of Helsinki with respect to human research and was approved by the ethics committee of The Second Affiliated Clinic Hospital (Xinqiao Hospital), Army Medical University (Third Military Medical University).

### The Echocardiography Examination

The echocardiographic examinations were performed by our skilled echocardiographist, Dr. Rongsheng Rao, according to the American Society of Echocardiography recommendations (19) in the morning after dialysis after been puncture to obtain fast venous blood samples. The patients were positioned in the supine position after a 10-min rest period while undergoing the echocardiography examination. Two-dimensional echocardiographically guided M-mode images were recorded from standardized views by using an ultrasonography system (CX50, Philips, USA) with a S5 probe. The end-diastolic internal diameters of the left atrium (LA), left ventricle (LVDD), right atrium (RA), right ventricle (RV), and pulmonary artery (PA) were measured. Furthermore, the interventricular septum (IVS) and its mobility, left ventricular posterior wall (LVPW) and its mobility, fractional shortening (FS), stroke volume (SV), and ejection fraction (EF) were also measured and recorded. In addition, tricuspid regurgitation (TR)-related parameters including TR area (TRA), TR velocity (TRV was used to calculate the PAP), and TR pressure were also examined.

### Biomarker Variable Determination

The patients' venous blood samples were collected in the early morning after fasting (>12 h). First, routine blood examinations were performed. The RBC count (RBC), mean corpuscular volume (MCV), hematocrit (HCT), hemoglobin concentration (Hb), red blood cell distribution width (RDW), white blood cell count (WBC), platelet (PLT) count, mean corpuscular hemoglobin concentration (MCHC), and mean corpuscular hemoglobin (MCH) were tested via an automated hematology corpuscle analyzer (AU400; Olympus Optical, Co., Tokyo, Japan). In addition, the lymphocyte ratio (LYM), monocyte ratio (MONO), neutrophil ratio (NEU), eosinophil ratio (EO), and basophil ratio (BAS) were calculated.

Next, plasma creatinine (Cr, enzyme assay) was also measured using Roche Diagnostics GmbH productions (Abbott, i2000, America). The sodium concentration (Na^+^) was determined by using indirect ion selective electrode assay (EX-Z, JOKOH, Japan). Tri-azo methods were used to measure the serum calcium concentration (Ca^2+^), while the phosphomolybdate ultraviolet method was employed to determine phosphate concentration (P) (Roche Diagnostics GmbH, America). In addition, the blood urea nitrogen concentration (BUN) and serum potassium concentration (K^+^) were also measured (FERENE methods, Beckman AU5821).

Each biochemical variable was measured from blood specimens in the Clinical Laboratory Department of the Second Affiliated Clinical College (Xinqiao Hospital).

### Variable Definitions

First, pulmonary artery systolic pressure (PASP) was calculated by the modified Bernoulli equation using the tricuspid systolic jet according to the recommendation by the American Society of Echocardiography: PASP = 4 × (TRV)^2^+ estimated right atrial pressure.

The estimated right atrial pressure was 5, 10, and 15 mmHg when the right atrium was normal, mildly enlarged, and significantly enlarged, respectively.

PH was defined as a PASP greater than 35 mmHg, while a PASP less than 35 mmHg is defined as normal non-PH patients according to the American Society of Echocardiography and other studies (19–22).

The severe increased PASP group is confirmedly defined PASP is more than 50 mmHg while PASP is between 36 and 50 mmHg is considered as mild increased PASP, other patients have been included into the normal PASP group according to the American Society of Echocardiography and other researches.

### Statistical Analysis

A continuous variable was described as the mean ± standard deviation (SD) if it was normally distributed. These variables were compared by using independent student's *t*-tests between non-PH and PH groups. The non-normally distributed variables are expressed as medians (25–75%), and these variables were compared with Non-parametric tests between PH and non-PH groups.

Univariate logistic regression analyses were primarily used to screen the associated factors for PH. A variable with a *p*-value < 0.05 in the univariate logistic regression was included in the multivariate (adjusted) logistic regression analyses to identify independent associated factors for PH. All of the statistical analyses were performed with the statistical software SPSS 22.0 (California, USA) for MAC.

## Results

### The Basic Information and Prevalence of PH in the Different Populations

**Figure 2:**
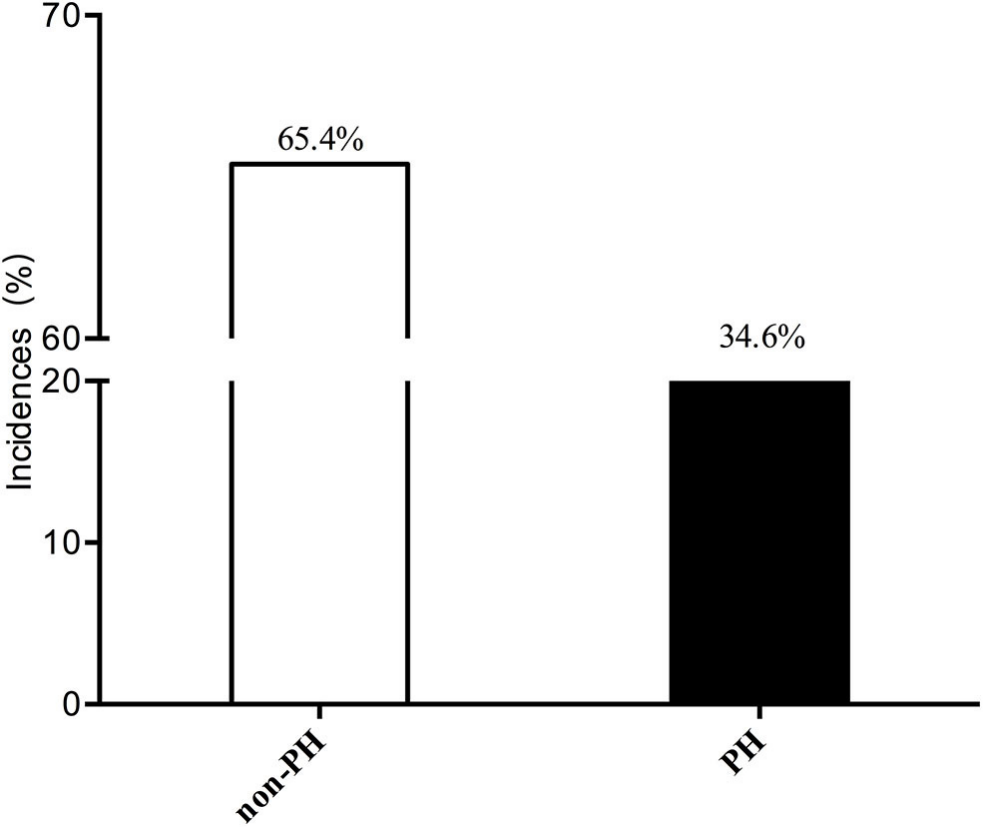
Incidence of PH in ESRD patients.

A total of 491 patients were included in our study. The mean age was 52.95 ± 14.21 years, and the mean BMI was 21.89 ± 4.89 kg/m^2^. The incidence of PH among ESRD patients who were treated with maintenance dialysis was 34.6% (170/491). Most of the patients had a normal PASP (65.4%, 321/491). These results are shown in Figure 2.

### The Differences in Cr and BUN, Echocardiographic, Blood Routine, and Electrolyte Parameters Between PH and non-PH Groups

In our present study, there were no differences in demographic data (age and BMI, *p* > 0.05) between the non-PH and PH groups.

Regarding the echocardiographic parameters, we first found differences in the structure and function of the left heart. We discovered that LA (42.06 ± 5.50 vs. 36.38 ± 4.56 mm) and LVDD (52.83 ± 6.71 vs. 47.73 ± 5.02 mm) were significantly higher in the PH group than in the normal PASP group (all *p*-values < 0.001, Table 1). The LVPW was significantly thinner in the PH group than that in the non-PH group between non-PH and PH groups (*p* =0.014), its mobility was more dispersive in the PH group than in the non-PH group (*p* < 0.001). However, the IVS was not different between the abovementioned two groups, and its mobility showed significantly more dispersion in the PH populations (all *p*-values < 0.001). The left heart function and LVEF were lower in the PH group (55.78 ± 12.06%) group than in the non-PH group (63.39 ± 7.26% *p* < 0.001). In accordance with LVEF, FS was lower in the PH group [22(28.0–35.0)], while it was significantly higher in the non-PH group [35.00 (32.25–37.00), *p* < 0.001]. However, the SV of the PH population was significantly higher than that of the non-PH group (*p* < 0.001). Regarding the right heart, RA (35.13 ± 3.42 vs. 41.05 ± 5.46 mm) and RV (34.01 ± 3.09 vs. 38.42 ± 4.83 mm) were significantly smaller in the non-PH patients than that in the PH ones (both *p* < 0.001, Table 1). Furthermore, the TR-related parameters, including TRA, TRV and ΔP, also revealed statistically significant differences between PH and non-PH groups (Table 1).

**Table 1:** Differences of the baseline parameters between PH and non-PH patients.

	**PH group (*n* = 111)**	**Non-PH group (*n* = 172)**	***p*-value**
**DEMOGRAPHIC DATA**			
Age (years)	52.42 ± 15.07	52.77 ± 13.79	0.844
BMI (kg/m^2^)	22.10 ± 3.35	22.66 ± 3.80	0.202
Gender (female, %)	38(34.2%)	69(40.1%)	0.380
**MEDICAL HISTORY**			
Hypertension (*n*, %)	74(66.7%)	110(64.0%)	0.799
ACEI/ARB	27(24.3%)	35(20.3%)	0.463
CCB	48(43.2%)	67(38.9%)	0.536
Diabetes (*n*, %)	19(17.1%)	25(14.5%)	0.615
**ECHOCARDIOGRAPHIC PARAMETERS**			
LA (mm)	42.06 ± 5.50	36.38 ± 4.56	<0.001^**^
LVDD (mm)	52.83 ± 6.71	47.73 ± 5.02	<0.001^**^
RA (mm)	41.05 ± 5.46	35.13 ± 3.42	<0.001^**^
RV (mm)	38.42 ± 4.83	34.01 ± 3.09	<0.001^**^
PA (mm)	24.90 ± 2.54	23.27 ± 2.32	<0.001^**^
IVS (mm)	12.48 ± 1.54	12.49 ± 1.67	0.951
IVS mobility	6.0(6.0–6.0)	6.0(6.0–6.0)	<0.001^**^
LVPW (mm)	12.0(10.0–12.4)	11.0(10.0–12.0)	0.014^*^
LVPW mobility	10.0(9.6–10.0)	10.0(10.0–10.0)	<0.001^**^
FS (%)	22(28.0–35.0)	35.00 (32.25–37.00)	<0.001^**^
LVEF (%)	55.78 ± 12.06	63.39 ± 7.26	<0.001^**^
SV(mL)	80.40 ± 21.66	74.40 ± 17.91	0.012^*^
TRA(cm^2^)	4.00(2.00–6.60)	1.00(1.00–1.88)	<0.001^**^
TRV (cm/s)	324.0(298.0–362.0)	245.00(228.75–255.00)	<0.001^**^
ΔP(mmHg)	42.0(35.0–53.0)	24.00(21.00–25.25)	<0.001^**^
**CR AND BUN**			
Cr (μmol/L)	814.40 (626.90–1019.30)	821.80 (613.38–1016.85)	0.799
BUN (μmoI/L)	21.90 ± 8.11	21.66 ± 7.66	0.805
**ELECTROLYTE PARAMETERS**			
Mg^2+^ (mmol/L)	1.000 ± 0.142	0.970 ± 0.167	0.123
K^+^ (mmol/L)	4.80 ± 0.83	4.79 ± 0.76	0.893
Ca^2+^ (mmol/L)	2.15 (1.98–2.31)	2.15 (2.04–2.28)	0.343
Na^+^(mmol/L)	137.8 (136.6–139.7)	137.9(136.2–140.0)	0.743
Cl^−^ (mmol/L)	103.60 (100.70–106.20)	102.15 (99.10–104.75)	0.016^*^
TCO_2_ (mmol/L)	20.21 ± 3.69	20.95 ± 4.30	0.136
P (mmol/L)	1.87 ± 0.64	1.94 ± 0.64	0.398
Dialysis time (days)	1314.0 (522.00–2635.0)	1216.0 (529.50–2300.50)	0.225

*BAS, basophil ratio; BUN, blood urea nitrogen concentration; Ca^2+^, serum calcium concentration; Cr, plasma creatinine; ESRD, end-stage renal disease; EO, eosinophil ratio; FS, fractional shortening; Hb, hemoglobin concentration; HCT, hematocrit; IVS, interventricular septum; K^+^, serum potassium concentration; LA, left atrium end-diastolic internal diameter; LVDD, left ventricle end-diastolic internal diameter; LVEF, ejection fraction of left ventricle; LVPW, left ventricular posterior wall; LYM, lymphocyte ratio; MCH, mean corpuscular hemoglobin; MCHC, mean corpuscular hemoglobin concentration; MCV, mean corpuscular volume; MONO, monocyte ratio; Na^+^, sodium concentration; NEU, neutrophil ratio; P, phosphate concentration; PA, pulmonary artery end-diastolic internal diameter; PAP, pulmonary arterial pressure; PASP, pulmonary artery systolic pressure; PH, pulmonary hypertension; PLT, platelet; RA, right atrium end-diastolic internal diameter; RBC, red blood cell count; RDW, red blood cell distribution width; RV, right ventricle end-diastolic internal diameter; SV, stroke volume; TR, tricuspid regurgitation; TRA, TR area; TRV, TR velocity; WBC, white blood cell count; ACEI,ARB, CCB*.

* *p < 0.05*,

** *p < 0.01*.

We further investigated the differences in Cr and BUN among the three groups. However, we did not find any differences in Cr or BUN between the PH and non-PH populations (all *p*-values > 0.05). Similarly, regarding electrolytes, the levels of Mg^2+^, K^+^, Ca^2+^, Na^+^, TCO_2_, and P were not different between the two groups. However, we found that the PH patients were characterized with a higher Cl^−^ concentration [103.60 (100.70–106.20) vs. 102.15 (99.10–104.75), *p* = 0.016, Table 1].

Finally, we examined the differences in routine blood examination parameters. The RBC and Hb-related parameters, RBC, MCV, HCT, MCH, MCHC, RDW-CV, and RDW-SD showed no differences PH and non-PH groups (all *p*-values > 0.05). In addition, the WBC and PLT count were not significantly different between the two groups mentioned above. The ratios of the other cell types, including the EO, BASO, MONO, NEU, and LYMP, in the PH group did not differ from non-PH group (all *p*-values > 0.05, Supplementary Table 1).

Furthermore, we have also compared the abovementioned parameters among normal PASP, mild increased PASP and severe increased PASP groups. The results have been listed in Supplementary Table 2.

### Correlations Between PASP and Demographic, Echocardiographic, Blood Routine, and Electrolyte Parameters as Well as Cr and BUN

We further analyzed the associations between PASP and demographic, echocardiographic, blood routine, and electrolyte parameters as well as Cr and BUN. First, regarding the demographic data, neither age (*r* = −0.034, *p* = 0.637) nor BMI (*r* = −0.052, *p* = 0.385) were correlated with PASP. Similarly, the dialysis time showed no association with PASP (*r* = 0.098, *p* = 0.101).

Importantly, we found that most of the echocardiographic parameters were associated with PASP. The structures of both the left and right heart, including LA (*r* = 0.549, *p* < 0.001), LVDD (*r* = 0.515, *p* < 0.001), FS (*r* = −0.323, *p* < 0.001), RA (*r* = 0.600, *p* < 0.001), and RV (*r* = 0.588, *p* < 0.001), showed a statistically significant relationship with PASP. Furthermore, the functions of the left heart, including LVEF (*r* = −0.421, *p* < 0.001) and SV (*r* = 0.158, *p* < 0.027), as well as the mobility of IVS (*r* = −0.333, *p* < 0.001), were also significantly correlated with PASP. TR-related parameters, TRA (*r* = 0.640, *p* < 0.001), TRV (*r* = 0.996, *p* < 0.001), and ΔP (*r* = 0.997, *p* < 0.001), were also related to PASP.

In our study, neither Cr nor BUN were correlated with PASP (all *p*-values were greater than 0.05). However, regarding the serum sodium ion concentrations, we found that Mg^2+^ (*r* = 0.149, *p* = 0.037) and Cl^−^ (*r* = 0.144, *p* = 0.043) were significantly associated with PASP. None of the following ion concentrations showed statistical associations with PASP: K^+^, Ca^2+^, Na^+^, TCO_2_, or P (all of the *p*-values were greater than 0.05, Table 2).

**Table 2:** Relationship between PASP and other parameters.

	**Relationship with PASP**	
**Parameters**	***r***	***p*-value**
**Demographic data**		
Age (years)	−0.034	0.637
BMI (kg/m^2^)	−0.052	0.385
**ECHOCARDIOGRAPHIC PARAMETERS**		
LA (mm)	0.549	<0.001
LVDD (mm)	0.515	<0.001
RA (mm)	0.600	<0.001
RV (mm)	0.588	<0.001
PA (mm)	0.383	<0.001
IVS (mm)	0.026	0.714
IVS mobility	−0.333	<0.001
LVPW (mm)	0.116	0.104
FS (%)	−0.323	<0.001
LVEF (%)	−0.421	<0.001
SV(mL)	0.158	0.027
TRA(cm^2^)	0.640	<0.001
TRV (cm/s)	0.996	<0.001
ΔP(mmHg)	0.997	<0.001
**CR AND BUN**		
Cr (μmol/L)	−0.007	0.921
BUN (μmoI/L)	−0.012	0.864
**ELECTROLYTE PARAMETERS**		
Mg^2+^ (mmol/L)	0.149	0.037
K^+^ (mmol/L)	0.040	0.576
Ca^2+^ (mmol/L)	0.007	0.927
Na^+^(mmol/L)	−0.015	0.830
Cl^−^ (mmol/L)	0.144	0.043
TCO_2_ (mmol/L)	−0.110	0.125
P (mmol/L)	−0.008	0.927
Dialysis time (days)	0.098	0.101

*This table shows the Spearman's correlations between PASP and the related factors*.

Finally, neither the RBC-related parameters nor WBC-related parameters showed any correlations with PASP. Additionally, neither the PLT count nor the ratios of other cell types were correlated with PASP (all *p*-values > 0.05, Supplementary Table 3).

### Logistic Regression for PH Using Demographic, Echocardiographic, Blood Routine, and Electrolyte Parameters as Well as Cr and BUN

First, we performed univariate logistic regression for PH using each variable. Neither age nor BMI showed any association with PH.

In the univariate logistic regression analysis, we found that LA, LVDD, RA, RV, PA, FS, IVS mobility, and LVEF were screened to be potential associated factors for PH (all of the *p*-values were less than 0.001, Table 3). Additionally, SV (OR: 1.016, 95%CI: 1.003–1.027, *p* = 0.013), LVPW (OR: 1.100, 95%CI: 1.028–1.399, *p* = 0.021), and its mobility (OR: 0.693, 95%CI: 0.567–0.848, *p* < 0.001) were also potential associated factors for PH.

**Table 3:** Univariate logistic analysis for PH with non-PH patients.

	**β**	***p*-value**	**OR**	**95CI%**	
**Parameters**				**Lower borderline**	**Upper borderline**
**DEMOGRAPHIC DATA**					
Age (years)	−0.002	0.843	0.998	0.982	1.015
BMI (kg/m^2^)	−0.044	0.202	0.957	0.895	1.024
**ECHOCARDIOGRAPHIC PARAMETERS**					
LA (mm)	0.239	<0.001	1.270	1.191	1.355
LVDD (mm)	0.148	<0.001	1.159	1.106	1.302
RA (mm)	0.309	<0.001	1.362	1.258	1.475
RV (mm)	0.301	<0.001	1.351	1.241	1.471
PA (mm)	0.277	<0.001	1.319	1.181	1.473
IVS (mm)	−0.005	0.951	0.958	0.858	1.154
IVS mobility	−0.563	<0.001	0.570	0.422	0.769
LVPW (mm)	0.182	0.021	1.100	1.028	1.399
LVPW mobility	−0.366	<0.001	0.693	0.567	0.848
FS (%)	−0.125	<0.001	0.883	0.843	0.924
LVEF (%)	−0.086	<0.001	0.917	0.889	0.946
SV(mL)	0.016	0.013	1.016	1.003	1.027
TRA(cm^2^)	1.227	<0.001	3.410	2.511	4.633
Dialysis time (days)	<0.001	0.192	1.000	1.000	1.000
**CR AND BUN**					
Cr (μmol/L)	0.000	0.717	1.000	0.999	1.001
BUN (μmoI/L)	0.004	0.804	1.004	0.974	1.035
**ELECTROLYTE PARAMETERS**					
Mg^2+^ (mmol/L)	1.9192	0.125	3.295	0.718	15.115
K^+^ (mmol/L)	−0.021	0.893	1.021	0.754	1.384
Ca^2+^ (mmol/L)	−0.363	0.477	0.695	0.255	1.893
Na^+^(mmol/L)	−0.033	0.423	0.968	0.893	1.048
Cl^−^ (mmol/L)	0.047	0.097	1.048	0.992	1.107
TCO_2_ (mmol/L)	−0.045	0.136	0.956	0.900	1.014
P (mmol/L)	−0.163	0.397	0.849	0.583	1.239
**BLOOD ROUTINE EXAMINATION PARAMETERS**					
RBC (10^12^ /L)	0.086	0.610	1.090	0.783	1.517
Hb (g/dL)	0.005	0.411	1.005	0.993	1.018
Hct (L/L)	0.018	0.362	1.018	0.979	1.059
MCV(fl)	−0.010	0.512	1.010	0.980	1.041
MCH (pg)	−0.019	0.681	1.019	0.932	1.114
MCHC(g/dL)	−0.004	0.663	0.996	0.978	1.014
RDW-CV (%)	0.043	0.559	1.044	0.904	1.206
RDW-SD (%)	0.018	0.289	1.019	0.985	1.054
PLT (10^9^/L)	−0.003	0.159	0.997	0.994	1.001
WBC (10^9^/L)	−0.047	0.363	0.954	0.862	1.056
EO (%)	−0.013	0.674	0.988	0.932	1.047
LYMP (%)	−0.008	0.644	0.992	0.960	1.026
BASO (%)	0.096	0.776	1.101	0.568	2.134
MONO (%)	0.029	0.527	1.030	0.940	1.154
NEU (%)	0.005	0.724	1.005	0.980	1.030

Furthermore, TRA (OR: 3.410, 95%CI: 2.511–4.633, *p* < 0.001) was also associated with PH.

Among the various ions, only Cl^−^ (OR: 1.048, 95%CI: 0.992–1.107, *p* = 0.097) showed associations with PH.

Finally, from the routine blood examination, none of them was included in the adjusted regression.

In the adjusted regression, we found that only LA (OR: 1.132, 95%CI: 1.047–1.224, *p* = 0.002), RA (OR: 1.236, 95%CI: 1.126–1.357, *p* < 0.001), and LVEF (OR: 0.945, 95%CI: 0.894–0.999) were independently associated with PH (Table 4).

**Table 4:** Adjusted logistic regressions for PH.

	**β**	***p*-value**	**OR**	**95CI%**	
**Parameters**				**Lower borderline**	**Upper borderline**
LA	0.124	0.002	1.134	1.048	1.225
RA	0.212	<0.001	1.236	1.126	1.357
LVEF	−0.056	0.046	0.945	0.894	0.999

## Discussion

In the present study, we found that PH is prevalent, with a relatively high incidence of 34.6% among 491 ESRD patients who undergo maintenance dialysis. We also investigated the associations between PASP/PH and Cr, BUN, serum ions, and routine blood parameters. Finally, we showed that only cardiac structure parameters, namely, LA, RA, and LVEF were independently associated with PH.

### PH Is Prevalent Among ESRD Patients Who Undergo Dialysis Treatment

PH is one of most frequent complications of numerous diseases, such as left heart failure, systemic lupus erythematosus (SLE) and renal dysfunction (1, 3, 16, 23). PH has been classified into two categories (primary PH or secondary PH) based on the presence of causes or risk factors (1). In ESRD patients, PH may be caused by the side effects of renal dysfunction (2). Furthermore, in many disease contexts, PH is considered one of the leading causes of death due to the associated complication of right heart failure (16, 24, 25). Thus, data on the prevalence of PH or PASP among dialysis patients are valuable toward informing guidelines for the management of complications in ESRD patients who undergo hemodialysis. In our present study, we found that PH occurs in maintenance dialysis ESRD patients. Previous studies have reported that the incidence of PH is high among ESRD patients, being as high as 39% among patients with stage 5 CKD and the incidence of PH is almost 68.8% among hemodialysis patients (9, 10, 16, 23, 26). PH is the basic physiopathologic process of many fatal diseases such as severe right heart failure and pulmonary edema (15).

In particular, some of the patients in this study showed a significantly higher PASP, reaching 90 mmHg, which requires further examination and treatment (to the outpatient department of cardiovascular diseases). The incidence of PH is similar to those of previous studies of hemodialysis patients. However, other meta-analysis-based studies have reported a higher incidence of PH in maintenance dialysis (hemodialysis and peritoneal dialysis). The incidence of PH in maintenance dialysis patients may be higher than that in the general population, and this difference may be due to Cr and BUN (26).

The patients who have been diagnosed with PH by the non-invasive method will be followed up in cardiovascular diseases department, they may need right heart catheterization examination and treatment.

### Associations Between PASP and Electrolytes Parameters, Cr and BUN

As discussed above, in ESRD patients, PH may be caused by imbalances in BUN, Cr and other toxins (including macromolecule toxins) that are induced by renal dysfunction (7, 25, 27). Furthermore, excessive serum ions also play adverse roles in PH (25). Thus, we examined the associations between PASP/PH and Cr and BUN as well as serum ions.

Previous studies have reported a relationship between PH and renal dysfunction (4, 13). Renal dysfunction is thought to contribute to the development of PH (2). However, we found that there were no differences in BUN or Cr among the various PASP groups. In addition, these parameters were not closely correlated with PASP.

It has been demonstrated that ions have various biological functions in the processes of cell proliferation, migration, and differentiation (28). In particular, K^+^ and Ca^2+^ have been reported to participate in pulmonary vasculature remodeling (29, 30). Thus, these ions may potentially play critical roles in PH. Our present study has also focused on the associations between ions and PASP/PH. However, in the first analysis, no significant differences were found among the three groups.

Emerging investigations of Mg^2+^ have focused on its effects on CREB proteins in neurons (31). In addition, its roles in cell proliferation and protein synthesis have been uncovered. However, the roles of Mg^2+^ in cardiovascular diseases have not been characterized completely. We tried to find an association between Mg^2+^ and PH, but instead, we found associations that need to be confirmed by further basic mechanistic studies.

### PH Has No Close Relationship With Routine Blood Parameters

Anemia is considered to be the most important complication in ESRD patients who undergo dialysis treatment (32). This is mainly caused by insufficient EPO production. It is also thought to lead to various diseases that may be caused by reductions in the oxygen supply, with consequent local and systemic hypoxia (32, 33). Thus, we also explored the associations between Hb- or RBC-related parameters and PASP or PH. However, we did not find any differences among the PASP groups. Additionally, these parameters were not closely related to PASP. Moreover, relationship between Hb- or RBC-related parameters and PH were not identified by further logistic regression analyses including the univariate and adjusted regressions. This may explain why the effects of Hb and RBC on PASP require a relatively long period of time. The lower Hb or RBC may induce hypoxia resulting in the proliferation of pulmonary artery vascular smooth muscle cells (34, 35).

We also examined the WBC- and PLT-count-related parameters as well as the ratios of other cells. However, no associations were identified.

### PH Is Independently Associated With RA, LA, and LVEF

Regarding the structure and function of the heart, PH patients had relatively high LA, RA, LVDD, and PA and relatively low left heart function. This finding is consistent with previous views on the structure and function of the heart in other cardiovascular diseases associated with PH (19, 36, 37). In the further analysis, only LA and RA were independently associated with PH. A large number of studies have shown that PH often accompanies changes in heart structure (19, 36, 37). The changes of right atrium may lead the secondary tricuspid regurgitation. The tricuspid regurgitation further increased the right atrium as a negative feedback. Both of the abovementioned conditions may induce the onset of PH. Thus, PH may be secondary to the enlargement of the right heart as well as its functions, leading to an enlarged tricuspid orifice or its relative insufficiency. Furthermore, the interactions between the left heart and right heart may also cause TR and PH. Many studies have indicated that changes in the structure and function of the left heart may lead to PH (38). Our study also found an association between LA and PH that warrants further cohort studies to confirm the role of the left heart in PH in the special subgroup of ESRD patients who undergo maintenance dialysis. The dialysis-influenced hemodynamics (left and right heart) may also be a reason for the development of PH. Thus, to inhabit the increase of left and right heart such as the use of Angiotensin-Converting Enzyme Inhibitors or Angiotensin Receptor Blockers may be a novel strategy to prevent PH. Finally, we found that the LVEF was closely associated with PH (may be showed a protective role). Thus, the future cohort study may be performed to identify the protective or predictive role of LVEF for PH.

## Limitations

Our present study was a retrospective study aimed at determining the prevalence of PH and its associated factors. There are several limitations in the study that may be improved in our future studies. First, it was a retrospective study that could identify only the associated factors for PH. The causality between clinical factors and PH/PASP should be determined by further cohort studies. Second, we included some of the frequently used parameters; however, there may be other more valuable factors that should receive attention. The state of nutrition, chronic inflammation and history have also been demonstrated to be associated with the onset of PH. Finally, pre-dialysis-associated parameters were not included in this study but will be included in our future studies.

## Conclusions

PH is prevalent, with a relatively high incidence among ESRD patients who undergo maintenance dialysis. The sizes of the left and right atria as well as LVEFF are independently associated with PH, but further cohort and basic mechanistic studies are needed to confirm this finding.

## Data Availability Statement

The raw data supporting the conclusions of this article will be made available by the authors, without undue reservation.

## Ethics Statement

The studies involving human participants were reviewed and approved by the ethics committee of The Second Affiliated Clinic Hospital (Xinqiao Hospital), Army Medical University (Third Military Medical University). The patients/participants provided their written informed consent to participate in this study.

## Author Contributions

S-ZB participated in the design of the study. YZ and S-ZB also drafted the manuscript and performed the statistical analysis. X-HD reviewed and revised this manuscript critically for important intellectual content. RR performed the echocardiography examination. RR performed the echocardiography examination and the analysis of PASP-related measurements. YZ and ST carried out the collection of demographic data and routine blood examination data. The other laboratory measurements were performed by YW and FP. The dialysis-related data were obtained by YZ, YW, and LN. All authors contributed to the article and approved the submitted version.

## Conflict of Interest

The authors declare that the research was conducted in the absence of any commercial or financial relationships that could be construed as a potential conflict of interest.

## Abbreviations

BAS: basophil ratio
BUN: blood urea nitrogen concentration
Ca^2+^: serum calcium concentration
Cr: plasma creatinine
ESRD: end-stage renal disease
EO: eosinophil ratio
FS: fractional shortening
Hb: hemoglobin concentration
HCT: hematocrit
IVS: interventricular septum
K^+^: serum potassium concentration
LA: left atrium end-diastolic internal diameter
LVDD: left ventricle end-diastolic internal diameter
LVEF: ejection fraction of left ventricle
LVPW: left ventricular posterior wall
LYM: lymphocyte ratio
MCH: mean corpuscular hemoglobin
MCHC: mean corpuscular hemoglobin concentration
MCV: mean corpuscular volume
MONO: monocyte ratio
Na^+^: sodium concentration
NEU: neutrophil ratio
P: phosphate concentration
PA: pulmonary artery end-diastolic internal diameter
PAP: pulmonary arterial pressure
PASP: pulmonary artery systolic pressure
PH: pulmonary hypertension
PLT: platelet
RA: right atrium end-diastolic internal diameter
RBC: red blood cell count
RDW: red blood cell distribution width
RV: right ventricle end-diastolic internal diameter
SV: stroke volume
TR: tricuspid regurgitation
TRA: TR area
TRV: TR velocity
WBC: white blood cell count.
