# Supplementary material for: The Prevalence of Pulmonary Hypertension Among Maintenance Dialysis Patients With ESRD and Its Associated Factors: A Retrospective Study
Source: Front Med (Lausanne). 2020 Dec 4;7:570874. doi: 10.3389/fmed.2020.570874 (PMC7746851; doi:10.3389/fmed.2020.570874)
Supplement: Supplementary file 1 [file Table_1.DOCX]

Supplementary Tables

Table S1 Differences of routine blood examination parameters between PH and non-PH groups

|  | PH group  (n = 111) | Non-PH group  (n = 172) | p value |
| --- | --- | --- | --- |
| *Routine blood examination parameters* |  |  |  |
| RBC (10^12^ /L) | 3.41±0.78 | 3.37±0.68 | 0.612 |
| Hb (g/dl) | 101.70±20.13 | 99.76±18.96 | 0.412 |
| Hct (L/L) | 32.26±6.37 | 31.58±5.96 | 0.363 |
| MCV(fl) | 95.53±8.28 | 94.89±7.86 | 0.514 |
| MCH (pg) | 30.12±2.78 | 29.98±2.65 | 0.683 |
| MCHC(g/dl) | 315.31±13.51 | 315.99±12.67 | 0.664 |
| RDW-CV (%) | 14.64±1.66 | 14.52±1.65 | 0.561 |
| RDW-SD (%) | 51.40 (47.40 - 55.70) | 51.25 (46.90 - 55.00) | 0.459 |
| PLT (10^9^/L) | 152.0 (119.0 - 206.0) | 171.76 (127.50 - 212.75) | 0.092 |
| WBC (10^9^/L) | 6.25 (5.12 – 7.86) | 6.86 (5.29 - 8.42) | 0.083 |
| EO (%) | 2.9(1.60 - 4.60) | 2.65 (1.50 - 4.78) | 0.475 |
| BASO (%) | 0.50(0.30 -0.70) | 0.50(0.30 -0.70) | 0.504 |
| MONO (%) | 6.20(4.70- 7.60) | 6.00(4.50- 7.48) | 0.500 |
| NEU (%) | 71.68±9.08 | 71.27±9.84 | 0.726 |
| LYMP (%) | 17.52±7.19 | 17.94±7.28 | 0.644 |

Table S3 Differences of the baseline parameters among various PASP groups

|  | Overall | Normal PASP group | Increased PASP group | |  |
| --- | --- | --- | --- | --- | --- |
| Parameters | (n = 283) | (n = 172) | Mild increased  PASP group (n = 52) | Severe increased PASP  group (n = 59) | p value |
| *Demographic data* | | | | | |
| Age (years) | 52.63±14.28 | 52.77±13.79 | 51.36±15.96 | 53.36±14.32 | 0.751 |
| BMI (kg/m^2^) | 22.44±3.64 | 22.66±3.80 | 22.24±3.56 | 21.98±3.18 | 0.415 |
| *Echocardiographic parameters* | | | | | |
| LA(mm) | 38.61±5.67 | 36.38±4.56 | 40.62±5.27^**^ | 43.32±5.43^**##^ | < 0.001 |
| LVDD(mm) | 49.73±6.25 | 47.73±5.02 | 50.52±6.21^**^ | 54.86±6.52^**##^ | < 0.001 |
| RA(mm) | 37.45±5.21 | 35.13±3.42 | 38.81±4.20^**^ | 43.03±5.70^**##^ | < 0.001 |
| RV(mm) | 35.74±4.42 | 34.01±3.09 | 36.38±3.74^**^ | 40.20±4.99^**##^ | < 0.001 |
| PA(mm) | 23.91±2.54 | 23.27±2.32 | 24.433±2.20^**^ | 25.31±2.80^**^ | < 0.001 |
| IVS(mm) | 12.49±1.61 | 12.49±1.67 | 12.60±1.36 | 12.37±1.69 | 0.767 |
| IVS mobility | 6.0(6.0 - 6.0) | 6.0(6.0 - 6.0) | 6.0(6.0 - 6.0) | 6.0(4.0 - 6.0) ^**##^ | < 0.001 |
| LVPW (mm) | 11.0(10.0 -12.0) | 11.0(10.0 -12.0) | 11.95 (10.25 - 12.40) | 12.00(10.00 - 12.30) | 0.110 |
| LVPW mobility | 10.0(10.0 -10.0) | 10.0(10.0 -10.0) | 10.0(10.0 -10.0) | 10.0(7.0 -10.0) ^**##^ | < 0.001 |
| FS (%) | 34.0(31.0 - 37.0) | 35.00 (32.25 - 37.00) | 32.50 (30.25 - 35.75) ^**^ | 31.00(20.00- 34.00) ^**##^ | < 0.001 |
| LVEF (%) | 60.40±10.12 | 63.39±7.26 | 59.55±9.33^**^ | 52.46±13.23^**##^ | < 0.001 |
| SV(ml) | 76.75±19.65 | 74.40±17.91 | 77.81±21.26 | 82.68±21.93^**^ | 0.018 |
| TRA(cm^2^) | 2.00(1.00 - 4.50) | 1.00(1.00 -1.88) | 2.90 (1.63 - 420) ^**^ | 5.80(3.50 - 9.10) ^**##^ | < 0.001 |
| TRV (cm/s) | 283.0(247.0 – 333.0) | 245.00(228.75 – 255.00) | 295.50 (283.25 - 313.00) ^**^ | 361.00 (340.00 - 380.00) ^**##^ | < 0.001 |
| ∆P(mmHg) | 32.0(24.0 - 44.0) | 24.00(21.00 - 25.25) | 34.50 (32.00 - 39.00) ^**^ | 52.00 (46.00 - 57.00) ^**##^ | < 0.001 |
| Cr and BUN | | | | | |
| Cr (umol/L) | 815.50 (618.40 - 1016.90) | 821.80 (613.38 - 1016.85) | 809.15 (568.15 - 968.90) | 819.20 (632.600 - 1034.90) | 0.058 |
| BUN (μmoI/L) | 21.75±7.82 | 21.66±7.66 | 22.51±7.98 | 21.36±8.24 | 0.772 |
| *Electrolyte parameters* | | | | | |
| Mg^2+^ (mmol/L) | 0.982±0.158 | 21.66±7.66 | 0.997±0.145 | 1.002±0.140 | 0.301 |
| K^+^ (mmol/L) | 4.79±0.79 | 4.79±0.76 | 4.82±0.815 | 4.78±0.85 | 0.960 |
| Ca^2+^ (mmol/L) | 2.15 (2.03 - 2.28) | 2.15 (2.04 - 2.28) | 2.14 (1.99 - 2.24) | 2.15 (1.96 - 2.35) | 0.604 |
| Na^+^(mmol/L) | 137.85(136.400 - 139.800) | 137.87 (136.22 - 140.00) | 138.40 (137.100 - 139.95) | 137.40 (136.50- 139.30) | 0.163 |
| Cl^-^ (mmol/L) | 102.44 (99.600- 105.300) | 102.15 (99.10 - 104.75) | 103.90 (101.52 - 106.90) | 103.20 (100.10 - 105.80) | 0.191 |
| TCO_2_ (mmol/L) | 20.66±4.08 | 20.95±4.30 | 20.73±3.55 | 19.75±3.77 | 0.149 |
| P (mmol/L) | 1.91±0.64 | 1.94±0.64 | 1.86±0.60 | 1.88±0.67 | 0.687 |
| Dialysis time (days) | 1230.0 (541.00 - 2549.0) | 1216.0 (529.50 - 2300.50) | 1487.00 (550.00 - 2732.50) | 1218.00 (552.00 - 2632.00) | 0.257 |
| *Routine blood examination parameters* | | | | | |
| RBC (10^12^ /L) | 3.39±0.72 | 3.37±0.68 | 3.30±0.70 | 3.51±0.85 | 0.277 |
| Hb (g/dl) | 100.52±19.41 | 99.76±18.96 | 98.41±18.16 | 104.60±21.45 | 0.175 |
| Hct (L/L) | 31.85±6.12 | 31.58±5.96 | 31.17±5.79 | 33.22±6.75 | 0.140 |
| MCV(fl) | 95.14±8.02 | 94.89±7.86 | 95.75±9.04 | 95.34±7.63 | 0.779 |
| MCH (pg) | 30.03±2.70 | 29.98±2.65 | 30.27±3.00 | 29.98±2.58 | 0.778 |
| MCHC(g/dl) | 315.72±12.99 | 315.99±12.67 | 316.24±14.03 | 314.48±13.10 | 0.706 |
| RDW-CV (%) | 14.56±1.65 | 14.52±1.65 | 14.65±1.60 | 14.62±1.72 | 0.840 |
| RDW-SD (%) | 51.30 (47.00 - 55.40) | 51.25 (46.90 - 55.00) | 51.50 (47.02 - 55.52) | 51.10 (48.30 - 56.10) | 0.532 |
| PLT (10^9^/L) | 167.0 (124.0 - 211.0) | 171.76 (127.50 - 212.75) | 161.00 (117.50 - 223.50) | 151.00 (126.00 - 200.00) | 0.135 |
| WBC (10^9^/L) | 6.61 (5.22 - 8.180) | 6.86 (5.29 - 8.42) | 5.98 (4.98 - 8.35) | 6.46 (5.12 - 7.07) | 0.315 |
| EO (%) | 2.80(1.60 - 4.70) | 2.65 (1.50 - 4.78) | 2.55 (1.50 - 4.075) | 3.50 (1.90 - 5.20) | 0.103 |
| BASO (%) | 0.050(0.030 -0.070) | 0.050(0.030 -0.070) | 0.450 (0.200 - 0.700) | 0.531 (0.300 - 0.800) | 0.335 |
| MONO (%) | 6.00(4.60- 7.50) | 6.00(4.50- 7.48) | 6.25 (4.42 - 7.47) | 6.10 (4.90- 7.70) | 0.425 |
| NEU (%) | 71.43±9.54 | 71.27±9.84 | 73.17±8.63 | 70.37±9.34 | 0.288 |
| LYMP (%) | 17.77±7.23 | 17.94±7.28 | 16.75±6.43 | 18.21±7.79 | 0.512 |

BAS: basophil ratio; BUN: blood urea nitrogen concentration; Ca^2+^: serum calcium concentration; Cr: plasma creatinine; eGFR: estimated glomerular filtration rate; ESRD: end-stage renal disease; EO: eosinophil ratio; FS: fractional shortening; Hb: hemoglobin concentration; HCT: hematocrit; IVS: interventricular septum; K^+^: serum potassium concentration; LA: left atrium end-diastolic internal diameter; LVDD: left ventricle end-diastolic internal diameter; LVEF: ejection fraction of left ventricle; LVPW: left ventricular posterior wall; LYM: lymphocyte ratio; MCH: mean corpuscular hemoglobin; MCHC: mean corpuscular hemoglobin concentration; MCV: mean corpuscular volume; MONO: monocyte ratio; Na^+^: sodium concentration; NEU: neutrophil ratio; P: phosphate concentration; PA: pulmonary artery end-diastolic internal diameter; PAP: pulmonary arterial pressure; PASP: pulmonary artery systolic pressure; PH: pulmonary hypertension; PLT: platelet; RA: right atrium end-diastolic internal diameter; RBC: red blood cell count; RDW: red blood cell distribution width; RV: right ventricle end-diastolic internal diameter;

SV: stroke volume; TR: tricuspid regurgitation; TRA: TR area; TRV: TR velocity; WBC: white blood cell count.

* compared with normal PASP < 0.05; ** compared with normal PASP < 0.01; # compared with Mild increased PASP < 0.05; ## compared with Mild increased PASP < 0.01.

Table S3 Relationship between PASP and *Routine blood examination parameters*

|  | Relationship with PASP | |
| --- | --- | --- |
| Parameters | r | p value |
| *Routine blood examination parameters* |  |  |
| RBC (10^12^ /L) | 0.102 | 0.152 |
| Hb (g/dl) | 0.120 | 0.092 |
| Hct (L/L) | 0.027 | 0.711 |
| MCV(fl) | -0.036 | 0.616 |
| MCH (pg) | -0.096 | 0.179 |
| MCHC(g/dl) | 0.052 | 0.470 |
| RDW-CV (%) | 0.054 | 0.452 |
| RDW-SD (%) | -0.095 | 0.185 |
| PLT (10^9^/L) | -0.138 | 0.054 |
| WBC (10^9^/L) | -0.029 | 0.689 |
| EO (%) | 0.082 | 0.249 |
| BASO (%) | 0.069 | 0.336 |
| MONO (%) | 0.037 | 0.608 |
| NEU (%) | 0.003 | 0.965 |
