# Supplementary figures and images for: The Prevalence of Pulmonary Hypertension Among Maintenance Dialysis Patients With ESRD and Its Associated Factors: A Retrospective Study
Source: Front Med (Lausanne). 2020 Dec 4;7:570874. doi: 10.3389/fmed.2020.570874 (PMC7746851; doi:10.3389/fmed.2020.570874)

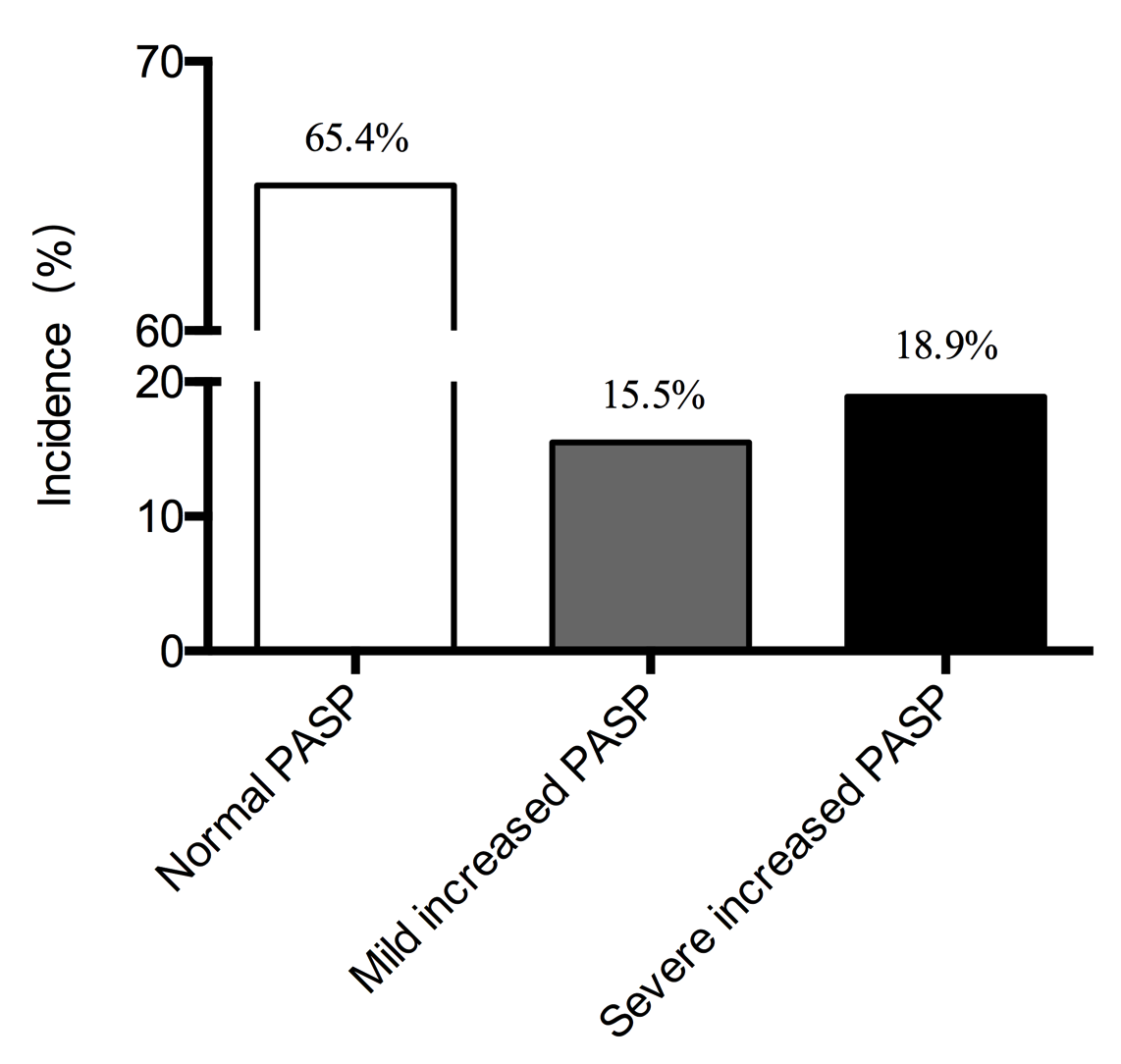

Supplement: Supplementary file 2 [file Image_1.TIFF]
